# Extraction of Nucleolus Candidate Zone in White Blood Cells of Peripheral Blood Smear Images Using Curvelet Transform

**DOI:** 10.1155/2012/574184

**Published:** 2012-05-15

**Authors:** Ramin Soltanzadeh, Hossein Rabbani, Ardeshir Talebi

**Affiliations:** ^1^Biomedical Engineering Department, Medical Image and Signal Processing Research Center, Isfahan University of Medical Sciences, Isfahan, Iran; ^2^Department of Pathology, School of Medicine, Isfahan University of Medical Sciences, Isfahan, Iran

## Abstract

The main part of each white blood cell (WBC) is its nucleus which contains chromosomes. Although white blood cells (WBCs) with giant nuclei are the main symptom of leukemia, they are not sufficient to prove this disease and other symptoms must be investigated. For example another important symptom of leukemia is the existence of nucleolus in nucleus. The nucleus contains chromatin and a structure called the nucleolus. Chromatin is DNA in its active form while nucleolus is composed of protein and RNA, which are usually inactive. In this paper, to diagnose this symptom and in order to discriminate between nucleoli and chromatins, we employ curvelet transform, which is a multiresolution transform for detecting 2D singularities in images. For this reason, at first nuclei are extracted by means of *K*-means method, then curvelet transform is applied on extracted nuclei and the coefficients are modified, and finally reconstructed image is used to extract the candidate locations of chromatins and nucleoli. This method is applied on 100 microscopic images and succeeds with specificity of 80.2% and sensitivity of 84.3% to detect the nucleolus candidate zone. After nucleolus candidate zone detection, new features that can be used to classify atypical and blast cells such as gradient of saturation channel are extracted.

## 1. Introduction

**Figure 1:**
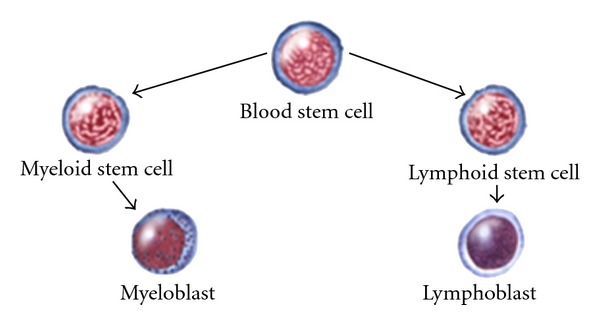
Different classes of human blood cells [2].

White blood cells (WBCs) or leukocytes play a significant role in the diagnosis of different diseases such as leukemia and different types of infections [1]; therefore, extracting information about them is valuable for hematologists. However, there are a few complications in extracting information from WBCs due to wide variation of these cells in shape, size, edge, and position. Generally, the WBCs are clustered in two major groups of myelogenic and lymphogenic [1] (Figure 1).

Leukemia is divided into two different categories of myeloblast and lymphoblast. These two groups are also divided into acute and chronic types. All leukemic cells are weak and cannot cooperate in the body defense procedure. By this form of clustering we have four types of leukemia which are called acute myelogenic leukemia (AML), chronic myelogenic leukemia (CML), acute lymphogenic leukemia (ALL), and chronic lymphogenic leukemia (CLL).

Clinically, these types have some common symptoms such as high nucleus to cytoplasm ratio (NCR), the size of nucleus, and existence of nucleolus, which is non-membrane bound structure inside nucleus, in nucleus [1].

 The use of image processing techniques in hematology has been developed rapidly in the last years, which helps hematologists to detect diseases automatically using blood smear images. These techniques can provide information about ratio of nucleus versus cytoplasm to identify and classify different types of WBCs such as neutrophile, basophile, lymphocyte, and so forth [3]. Many efforts have been done in the area of general segmentation of WBCs by means of methods such as edge and border detection, region growing, filtering, mathematical morphology, and watershed clustering [3]. Ritter and Cooper [4] presented an automatic method which segments and identifies the objects in a blood smear image. However their work has some restrictions like inability to find overlapped objects which is the reason why most of the detailed information has been lost in the images. Ongun et al. [5] segmented the objects in a microscopic image based on morphological preprocessing followed by iteration algorithms like snake edge detection. Jiang et al. [6] proposed WBCs segmentation by different methods like snake and watershed in color space. Dorini et al. [7] enhanced the segmentation accuracy by using scale-space operators. The automatic morphological method of Scotti [8] was based on the morphological analysis of WBCs and shape of normal cells. Their proposed system extracts the morphological indexes (lymphocytes). Kumar et al. [9] used Teager energy operator for nucleus segmentation with edges, which are detected effectively by Teager energy operator, but it was restricted because of low contrast between the gray level of cells and background [3]. For cytoplasm segmentation, they used a simple morphological method. Cseke used multilevel segmentation method [10], which used Otsu thresholding algorithm [11]. Recently, Ko et al. have used the gradient vector flow and snakes method which is based on probability density function estimated from samples of WBCs nuclei and the other parts of the images [12]. Moreover, many attempts on image enhancement have been done in this area to show the WBCs nucleus better; as a sample, Halim et al. used a global contrast stretching technique in order to increase the nucleus image quality in the HIS color system [13]. 

Another work, which was done on the fluorescence in situ hybridization images, was reported by Jeong et al. [14]. They used a threshold, which was estimated using a Gaussian mixture model and maximizing the likelihood function of the grey values for the cell images, to segment the cell nuclei from the background; then the overlapped and isolated nuclei are classified to facilitate a more accurate nuclei analysis. In order to do this pipeline, the morphological features of the nuclei, such as their compactness, smoothness, and moments, are extracted and applied to Bayesian networks for training [14]. As an illustration which used evolutionary methods, Yi et al. [15] have used online trained neural network as a classifier for segmentation of WBCs from the image and applied particle swarm optimization (PSO) algorithm for training their classifier in order to converge the training procedure faster [15]. Jiang and his colleagues have reported a novel segmentation which was based on watershed technique. This watershed clustering has been done in 3D HSV (hue-saturation-volume) color system. They use HSV color system because of low correlation between its different channels [16].

 Although the mentioned methods are able to generally segment WBCs, more advanced techniques are required for exploring inside WBCs. For example, beside giant nucleus as the main symptom of leukemia, another important symptom for this disease is the existence of nucleolus in nucleus. If we could extract nucleolus candidate zone, then we will be able to classify blast cells and atypical cells by additional processing steps. In fact atypical leukocytes have chromatins, with empty space and low saturation, which can be wrongly detected as nucleolus. The key point to classify blast cells and atypical ones is the answer of this question: “How much the texture of candidate zone for nucleoli is more hyper-chromic than the texture of nucleus?” [14]; that means the smaller the gradient, the more chance of it being a blast cell. The main reason is that blast cells have nucleoli that contain liquid; so, their saturation is not far from texture of nucleus [1, 14].

 In this study, at first, nuclei are extracted by clustering the microscopic images into three color clusters in Luv color system using *K*-means method [15, 16]. To extract candidate zone for nucleoli (the zone of both nucleoli and chromatins in nucleus), the curvelet transform [17] is applied on extracted nucleus. Curvelet transform is an appropriate transform for detecting detailed information in images due to its optimality for extraction of 2D singularities. This property makes this transform a near-optimal tool for extraction of 2D singularity-based features (such as curves in images) especially in case of investigating very small and detailed features (such as nucleoli in WBC images). So, after image reconstruction based on modified curvelet coefficients using a similar thresholding criterion suggested in [18] and some postprocessing steps the nucleolus candidate zone is extracted. After extraction of these regions, we find the color saturation gradient of nucleus texture and suggest a new feature for discrimination between atypical and blast cases. This feature guides us to classify the lymphoblast cells and atypical lymphoma cells.

The rest of this paper is organized as follows. In Section 2, the segmentation algorithm for nucleus extraction is explained. In Section 3, the proposed curvelet-based method for nucleolus detection is described.  Section 4 is dedicated to results, and finally, the conclusions are drawn in Section 5.

## 2. Nucleus Segmentation

**Figure 2:**
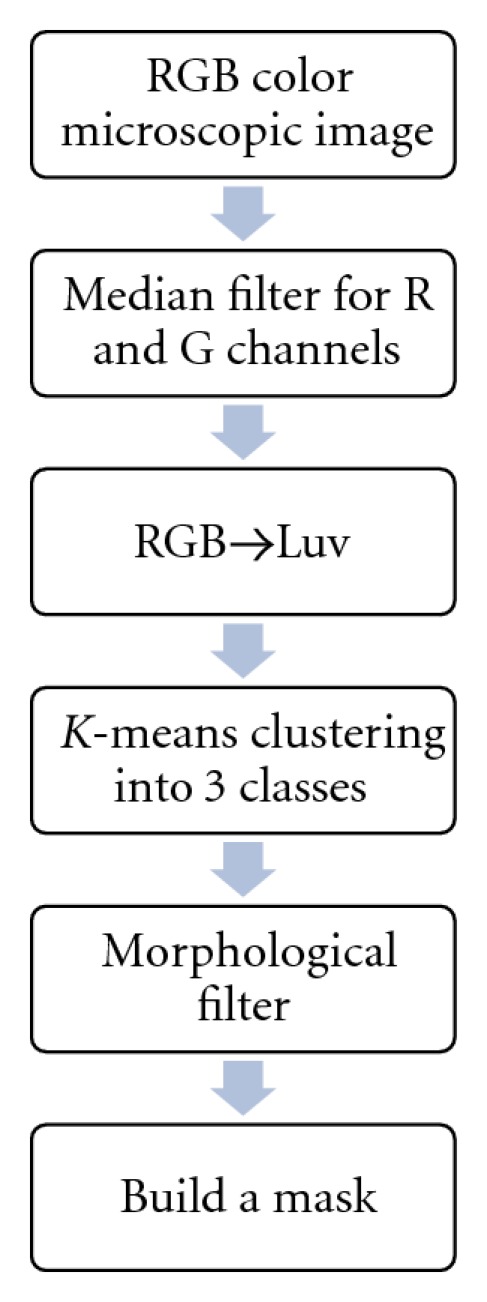
Nucleus segmentation procedure.

The aim of nucleus segmentation is separating the nucleus from the other parts of a cell and a microscopic blood smear image. A typical peripheral blood smear image consists of four components: red cells (unnucleated cells), white blood cells nucleus, cytoplasm, and background which contains platelets and even spot noise. Usually WBCs appear in a different color from red cells and the other parts of a peripheral blood smear images. In this section by using color information, after applying several preprocessing steps, nuclei are extracted by means of *K*-means method [19–21]. Till now, several methods have been reported for color image segmentation. For example in [19] a self-organizing map-based *K*-means method is proposed. In this paper we use a simple technique such as proposed method in [20].  Figure 2 shows the WBCs nucleus segmentation procedure.

**Figure 3:**
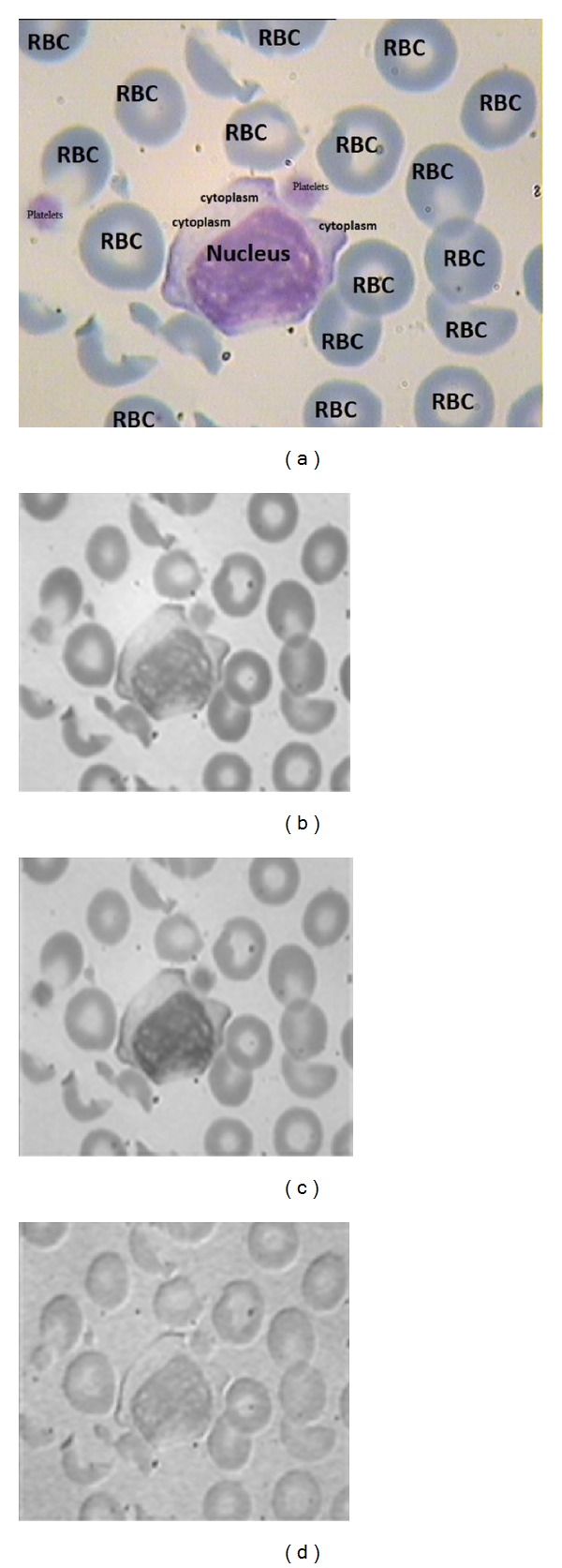
(a) A sample RGB microscopic image; (b) R channel; (c) G channel; (d) B channel.

 At first, the microscopic image is separated into R, G, and B components. The median filter is applied only on R and G to decrease spot noise and maintain the edge quality as much as possible. As shown in Figure 3, B channel has no specific data on nucleus. Because it has approximately same gray level of RBCs and background, the processing is continued only on R and G channels.

**Figure 4:**
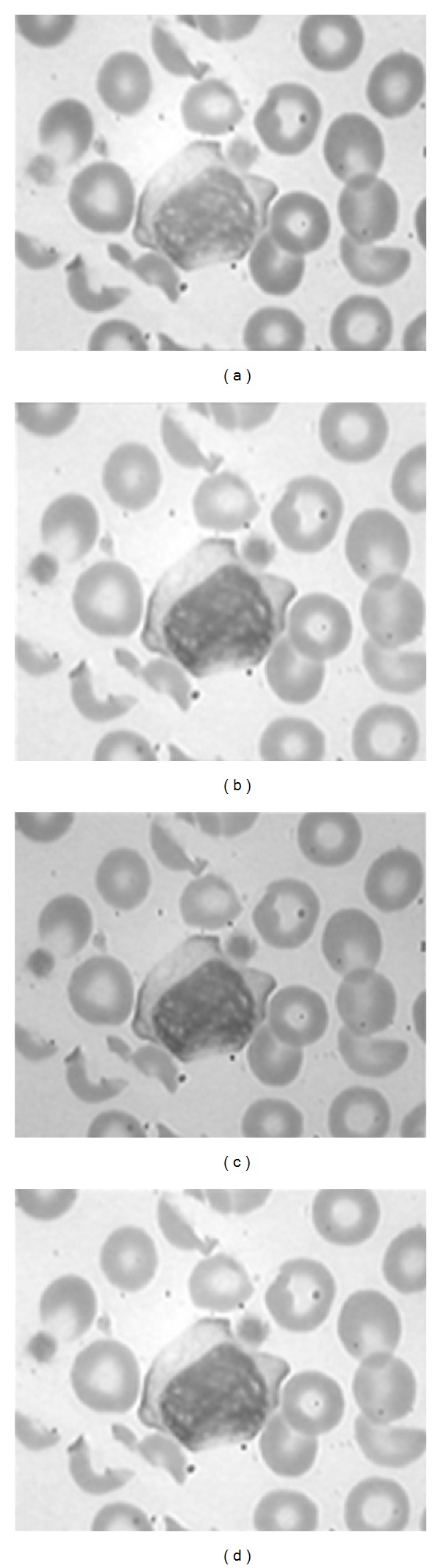
(a) Main R component; (b) output of applying median filter on R; (c) main G component; (d) output of applying median filter on G.

**Figure 5:**
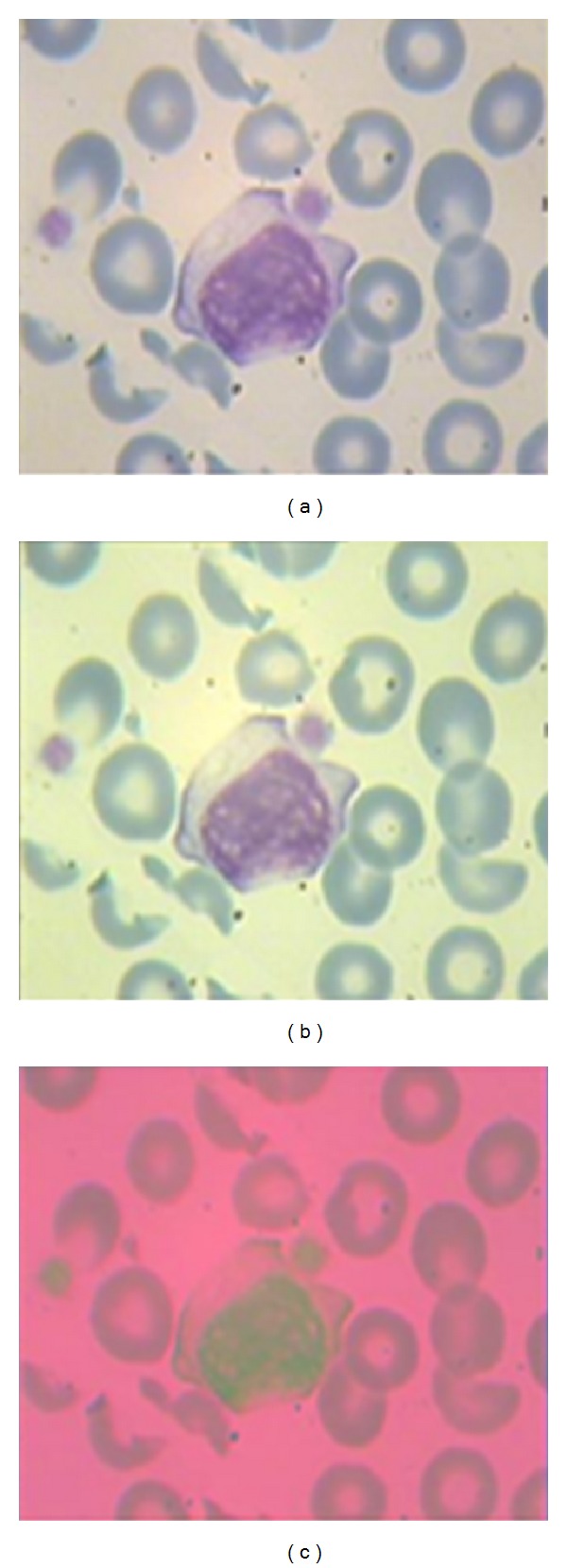
(a) Main RGB image; (b) enhanced RGB image; (c) Luv color system of enhanced RBG image.

The results of applying median filter and grayscale histogram equalization on R and G channels are shown in Figure 4. After that, the RGB image is reconstructed by filtered R and G components and the main B component. In this case, the the nucleus keeps its own color but the background of the image and RBCs become brighter. Now, we need some color system which can predominant the color of nucleus from the color of background and RBCs. This color system can be Luv color system which has three independent channels [22]. It has been shown that this color system specify the nucleus from the other objects of the image [23]. The dependency of color channels is decreased in LUV system rather than RGB because of more Euclidean distances.  Figure 5 shows the enhanced image and its LUV color system.

**Figure 6:**
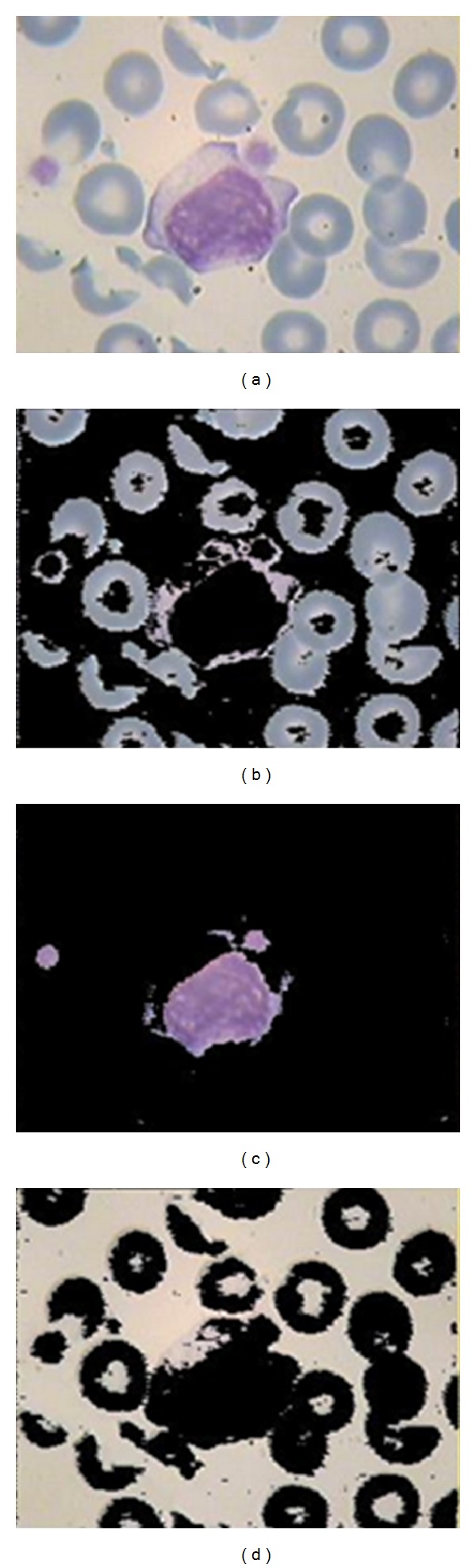
(a) Original image; (b) class 1 from color clustering; (c) class 2 from color clustering; (d) class 3 from color clustering.

**Figure 7:**
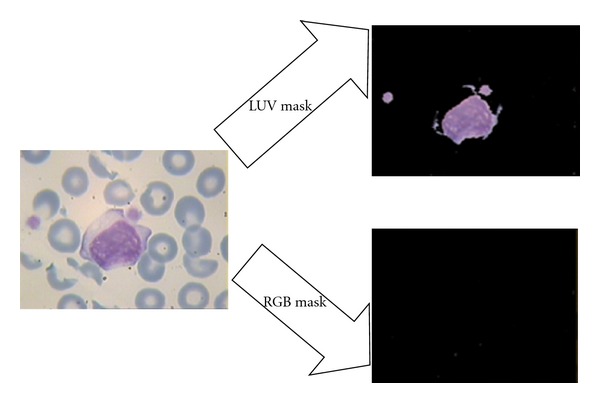
A comparisons between the results of applying *K-*means in RGB and Luv domains.

 It is clear from Figure 5 that this image representation system can discriminate the WBC nucleus from the other objects of image. Now, we can classify the colors of LUV system into 3 classes which can be done by applying a simple *K-*means clustering method on Luv color system and mapping this clusters again to RGB color system. These three classes are shown in Figure 6 in RGB color system. Note that applying *K-*means clustering method in RGB domain does not lead to an acceptable result (Figure 7).

**Figure 8:**
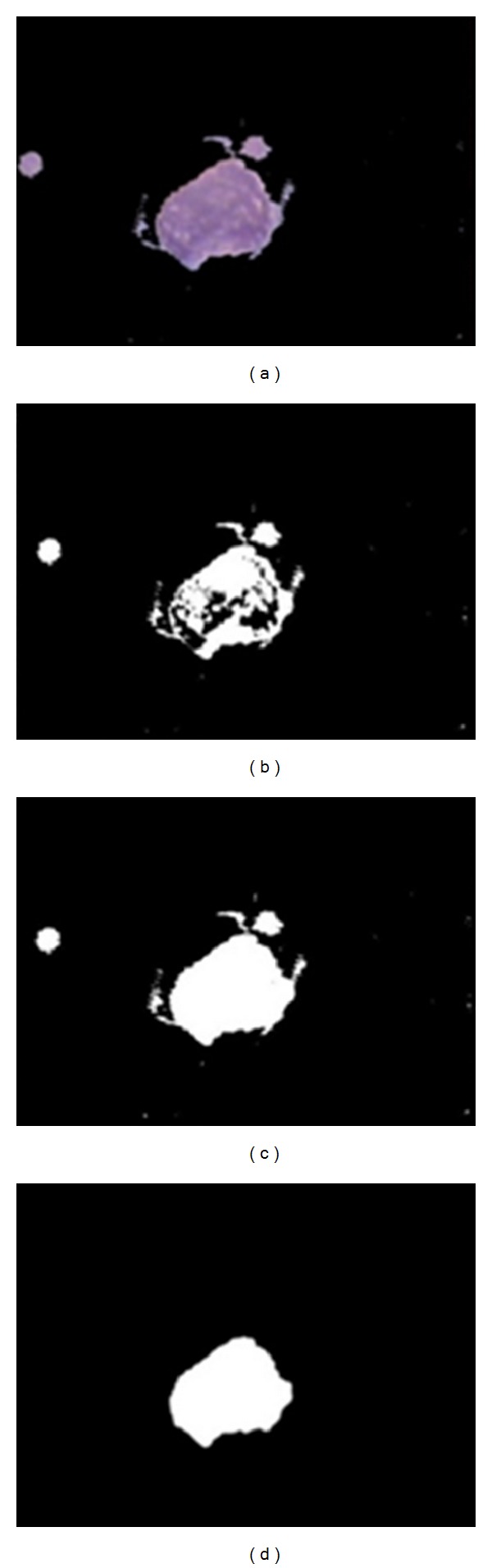
(a) The extracted nucleus region by applying *K-*means clustering; (b) the extracted binary mask from (a); (c) hole-filled binary mask of nucleus; (d) the final mask of the nucleus after applying area filters.

**Figure 9:**
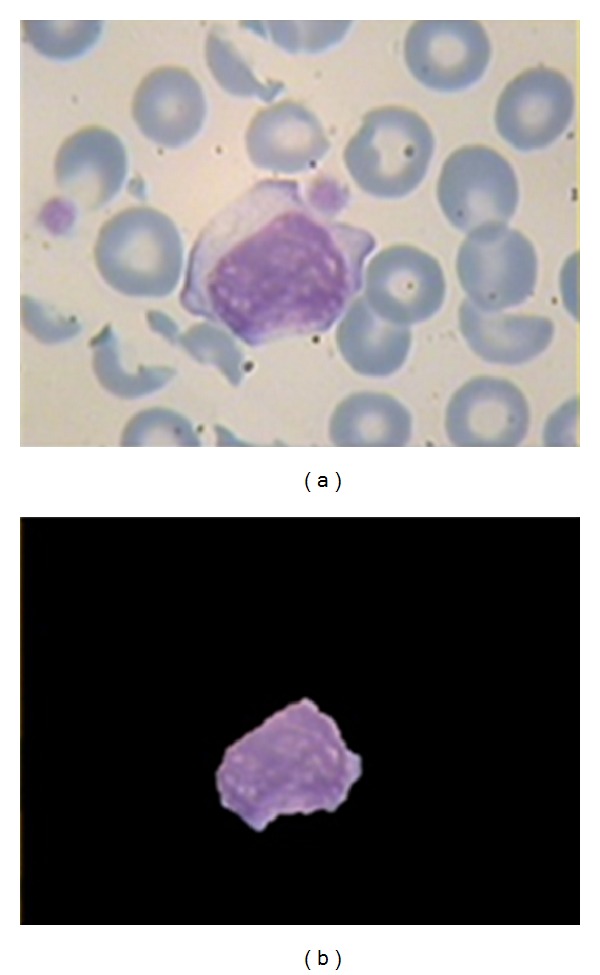
(a) Main image; (b) the result of applying extracted mask (in Figure 8) for nucleus extraction.

 To specify which image (in Figures 6(b), 6(c), and 6(d)) indicates the nucleus, the mean value of R channel is calculated for each image and the image with minimum value would be the candidate of nucleus. Then, the mask of nucleus is made by thresholding in grayscale image and after hole filling, we have a mask with extra white objects which must be omited from the nucleus mask. To eliminate the extra objects detected as nucleus, morphological opening (with 2 pixels diameter disk) and closing (with 8 pixels diameter disk) and area filters, which omit the white objects with area less than 5000 pixels, are applied. The area elimination number (5000 pixels) is chosen by trial and error due to this fact that any healthy or blast lymphocytes have a restricted size [24]. The results of these steps are shown in Figure 8.  Figure 9 shows the extracted nucleus of WBCs obtained by applying the extracted mask on Figure 8.

**Figure 10:**
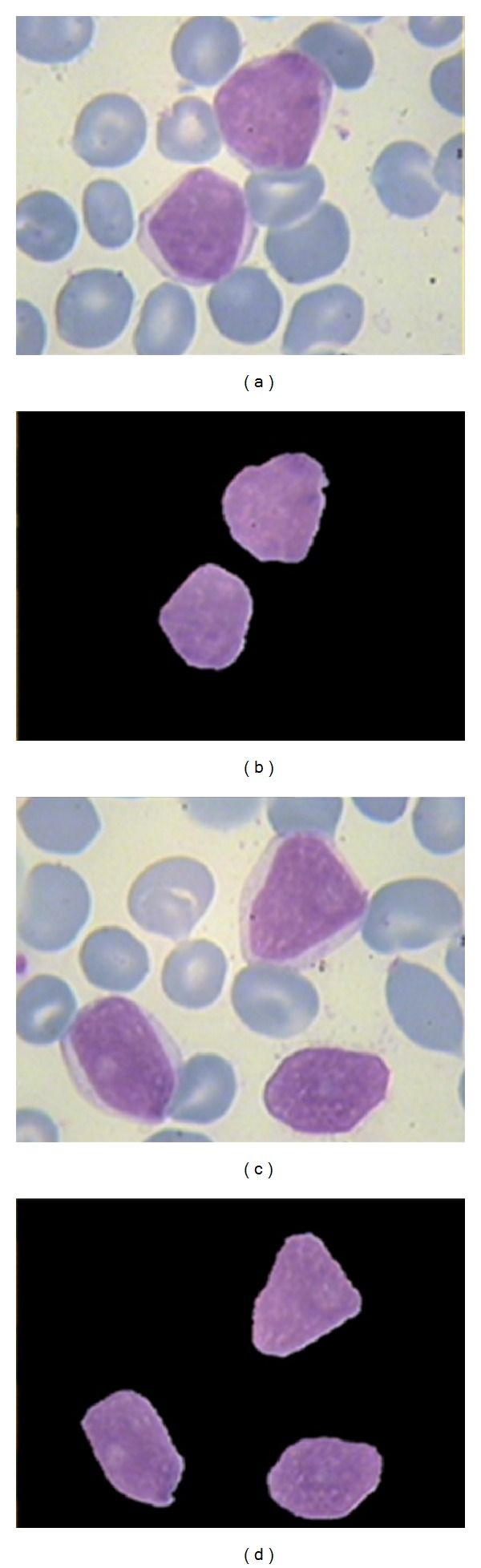
(a, c) Main image; (b, d) the result of applying extracted mask for 2 and 3 nucleus extraction.

Note that this method can also be applied to the images with more than one nucleus which is illustrated in Figure 10.

In addition, in some cases such as the case where the edge of nucleus be more transparent than other regions in nucleus, a bad segmentation result may be obtained. 

## 3. Extraction of Candidate Zone of Nucleolus

 In this section at first we introduce a method for detection of candidate zone that shows the candidate regions for either chromatins or nucleoli. In the next step we suggest a new feature based on the gradient of saturation channel by which we try to distinguish between chromatins and nucleoli. The results of this step can be used as a new feature for classification of blast and atypical cells.

### 3.1. Candidate Zone Detection

To extract nucleolus, the curvelet wrapping transform is applied to the extracted nucleus image (in previous section). In this level, the main image is decomposed to different subbands with different resolutions. To apply curvelet transform, the proposed algorithm in [25, 26] is used. This algorithm decomposes an *N* × *N* image as follows:

(1) $$\begin{array}{c} f\left(x,y\right)=c_{J}\left(x,y\right)+\overset{J}{\underset{j=1}{\sum }}w_{j}\left(x,y\right), \end{array}$$

where *c*_*J*_(*x*, *y*) shows the lowpass coefficients of image and *w*_*j*_ are the detail coefficients of the image in scale of 2^−*j*^. The output of this algorithm is composed of *J* + 1 subbands by the size of *N* × *N*.

**Figure 11:**
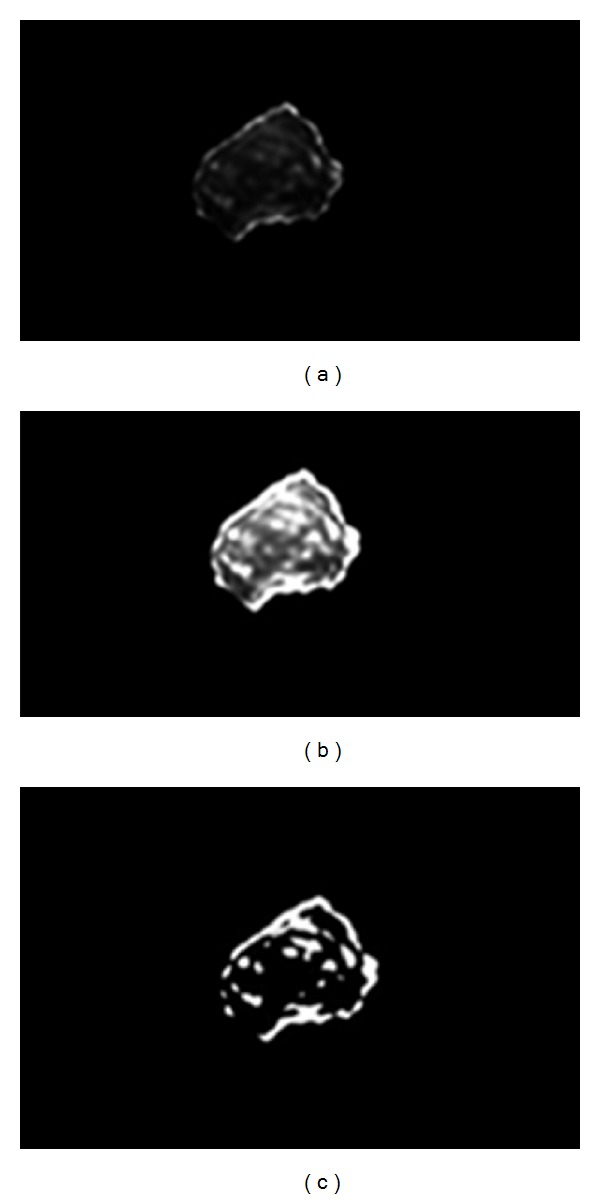
(a) Reconstructed image after modification of curvelet coefficients; (b) enhanced grayscale of (a) using stretching; (c) binary thresholding of (b).

**Figure 12:**
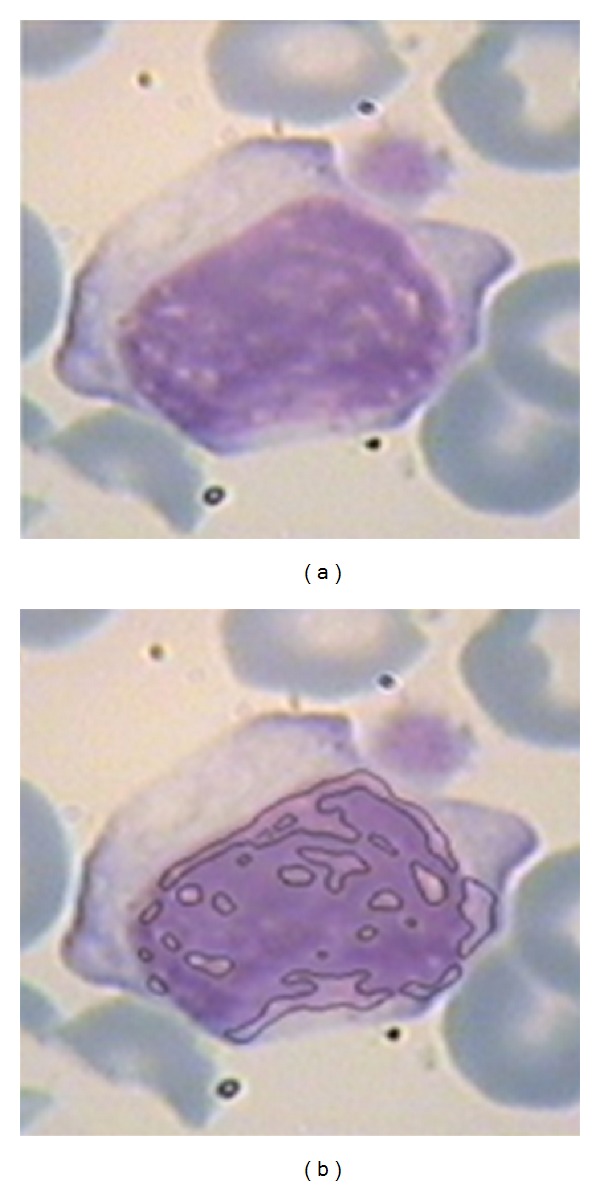
(a) Main image, (b) Extraction of candidate zone.

**Figure 13:**
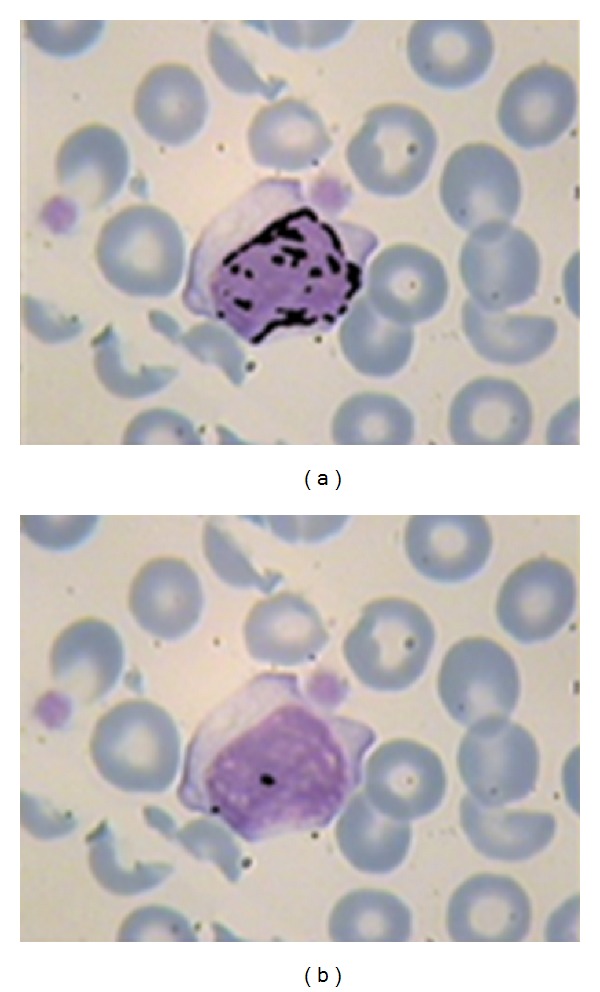
(a) Extracted candidate zone (black area) using “power ten” criterion; (b) extracted candidate zone (black area) using constant criterion in [25].

 In this study, wrapping curvelet transform [25] in 4 levels and 8 angles is applied on the extracted nucleus (using proposed method in previous section and using grayscale image) and then the coefficients are modified using the following modified threshold suggested in [25]

(2) $$y_{c}\left(x,\sigma \right)=\{\begin{array}{cc} x^{n}, & x<c\sigma ,\\ \frac{x-c\sigma }{c\sigma }{\left(\frac{m}{c\sigma }\right)}^{p}+\frac{2c\sigma -x}{c\sigma }, & c\sigma <x<2c\sigma ,\\ {\left(\frac{m}{x}\right)}^{p}, & 2c\sigma <x<m,\\ {\left(\frac{m}{x}\right)}^{s}, & x>m, \end{array}$$

where *y*_*c*_(*x*, *σ*) is the value of coefficient of output curvelet transform, *p* is the parameter of nonlinearity, and *s* is the dynamic range of each subband. *m* is a parameter which depends on the curvelet coefficient in each subband and is calculated by  (3)

(3) $$m=lM_{c},$$

where *M*_*c*_ is the maximum of each subband and *l* is between 0 and 1. In this study, the proposed parameters are *n* = 10, *l* = 0.01, *c* = 5, *p* = 2, and *s* = 10. It is considerable that these values are obtained experimentally and optimized by trial and error. After modification of details subbands, the coarse subband is multiplied to 10 in order to enhance the low-resolution information in the image and then the image is reconstructed. Then a simple grayscale stretching and Otsu grayscale thresholding [11] are applied on the image to make a mask of details of nucleus.  Figure 11 shows the results of these steps for extracted nucleus in previous step, and Figure 12 shows the results of applying extracted mask (in Figure 11) on main image. Note that in [25], the curvelet coefficients smaller than *cσ* are replaced with a constant. This constant can be the same for all of the subbands or be adaptive with the coefficients. As it has been shown in Figure 13, the “power ten” criterion used in this paper is able to extract the nucleolus candidate zone while using the constant criterion cannot. The main reason is that using “power ten” criterion we would be able to reduce low-frequency component and attenuate the soft area that leads to domination of details including nucleolus candidate zone. 

**Figure 14:**
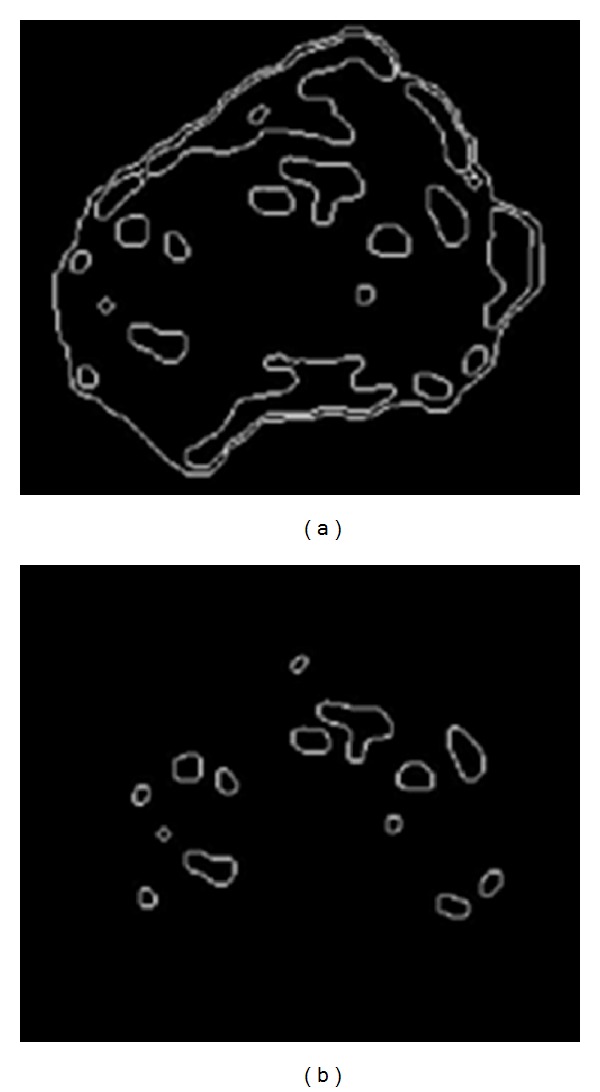
(a) All edges of Figure 11(c); (b) after removing false detected nucleoli from (a).

Note that the mentioned method detects all of the fine edges. To omit the edge of nucleus, a simple edge detection method like *canny *method is applied on the mask, and then this part of image is omitted by labeling to remove false detected nucleoli (Figure 14).

**Figure 15:**
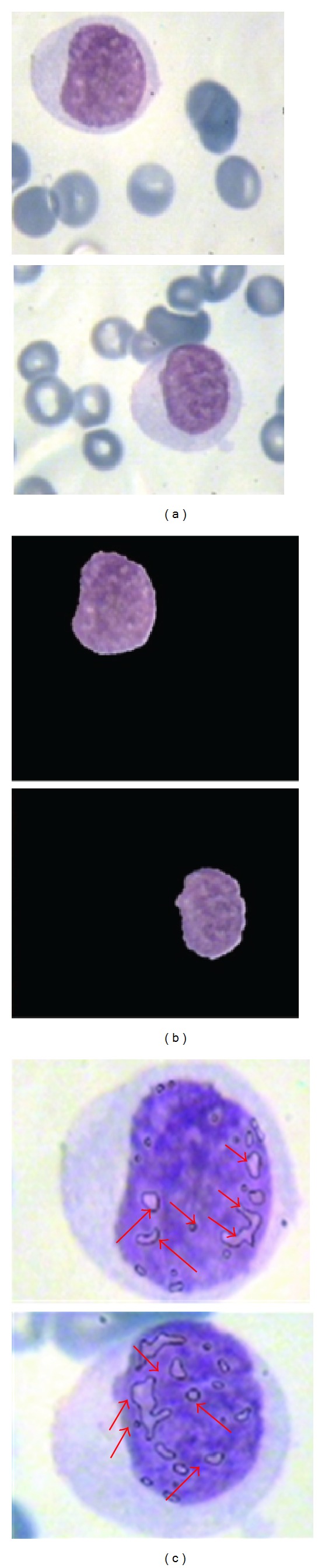
(a) Main image; (b) nucleus detection; (c) candidate zone detection.

In Figure 15 the results of applying our candidate zone extraction algorithm on several images have been shown.

### 3.2. A New Feature Based on the Gradient of Saturation Channel

By applying the extracted mask from candidate zone extraction step on S channel (in HSI color system), the gradient of color saturation (*G*) can be defined as follows:

(4) $$G=\text{mi}{\text{n}}_{\text{nucleus}\;\;\text{texture}}f\left(i,j\right)-\text{ma}{\text{x}}_{\text{candidate}\;\;\text{zone}}f\left(i,j\right).$$

**Figure 16:**
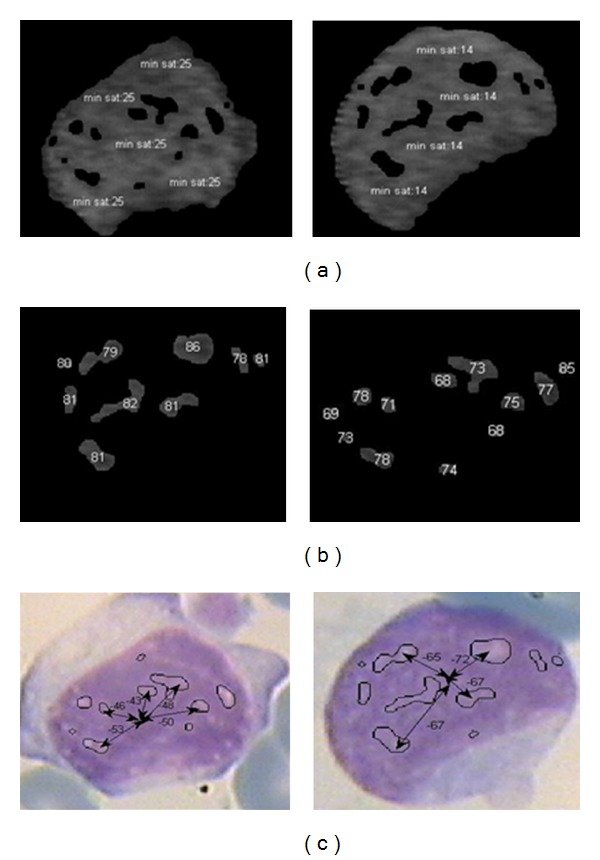
(a) Minimum saturation value of nucleus texture for an atypical cell (left images) and blast cell (right images); (b) maximum saturation of some candidate zones; (c) the gradient measure for some regions.

In (4), where *f*(*i*, *j*) is the value of S channel, the minimum value of S channel is subtracted from the maximum value of nucleoli saturation. The mean saturation level of each candidate zone of nucleoli is illustrated in Figure 16 (the maximum saturation value of each pixel is 255). Note that in this figure we have 14 distinct candidate regions whose mean gradient measure is calculated by averaging gradient measure of each region. As leukemic cells have usually fine nucleus texture [2], in this type of cells *G* is far from zero, but in atypical lymphoma cells this value is nearer to 0. The main reason is that nucleoli contain liquid in and their color is darker than chromatins created by different methods of chromosome folding [1, 27]. So, this parameter helps us to discriminate the chromatins from nuclei. Figure 16 shows some detailed results for extraction of gradient vectors, and Table 1 illustrates the final gradient values.

Our simulations show that mean of gradient (of saturation channel) is not an appropriate feature in all cases for distinguishing between atypical and blast cells and other features obtained from gradient measure, and/or other extracted information from the cell can be more useful for final blast cell detection. For example as shown in Table 1, the average of calculated gradient for an atypical cell is far from the maximum gradient measure; however this value (the difference between average and maximum gradient, namely, “maximum standard error”) is lower in blast cells. In other words, the maximum gradient of saturation channel of nucleoli candidate zones is near to average gradient of all candidate zones of nucleus, and this parameter can be also used for nucleus detection.

## 4. Results

In this section, the final results of nucleolus candidate zone detection are presented. This algorithm was tested on 100 microscopic images of size 768 × 576 captured with a simple light microscope with three ocular lenses and an analog video CCD which is coupled to a Pinnacle to digitize the captured images (these images are available on http://misp.mui.ac.ir/data/microscopic-image-data.html). The specificity and sensitivity of this method is calculated using the following formula:

(5) $$\begin{array}{c} \text{sensitivit}\;\;y=\frac{\text{TP}}{\text{TP}+\text{FN}},\\ \text{specificit}\;\;y=\frac{\text{TN}}{\text{TN}+\text{FP}}, \end{array}$$

where TP, FP, FN, and TN stand for true positive results, false positive results, false negative results, and true negative results. 

**Figure 17:**
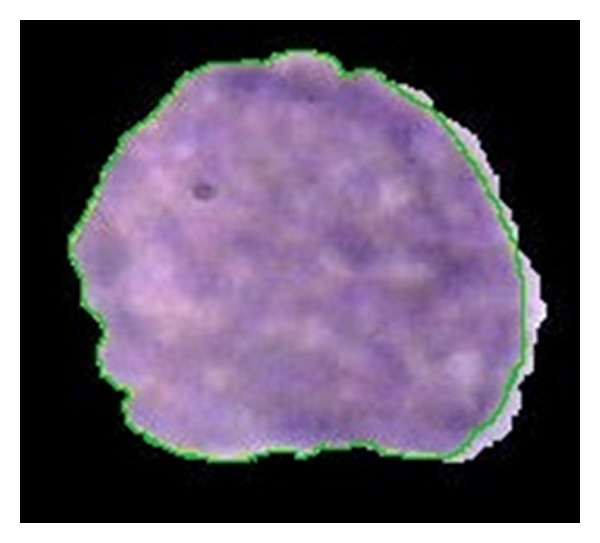
Comparison between extracted regions as nucleus by our algorithm (29838 pixels) and detected regions by pathologist illustrated by green edge (28346 pixels).

For nucleus detection, TP is the common area between regions extracted by algorithm and regions detected by the pathologist. FP is the area that does not belong to nucleus but our algorithm detects it as nucleus. TN is the area which does not belong to nucleus and our algorithm also does not detect that region as a part of nucleus. FN is the area that belongs to nucleus but our algorithm is not able to detect it as a part of nucleus.  Figure 17 shows a comparison between extracted regions as nucleus by our algorithm and detected regions by pathologist. In this figure, the area determined by pathologist is indicated with green line, and the detected region by our algorithm is surrounded by black area. 

**Figure 18:**
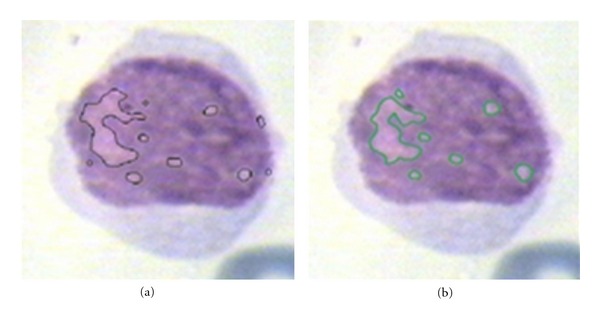
A comparison between candidate regions detected by pathologist (3052 pixels) and extracted area by our algorithm (3611 pixels). (a): extracted nucleoli by expert; (b): the results of our algorithm for nucleolus detection.

 Similarly for nucleolus candidate zone detection, TP is the area detected as candidate zone of nucleolus by our algorithm and pathologist and TN is the area not detected as candidate zone of nucleolus by our algorithm and pathologist. FP is the area detected as candidate zone of nucleolus by our algorithm but this area is not candidate zone of nucleolus based on pathologist's decision. Finally, FN is the area not detected as candidate zone of nucleolus by our algorithm but this area is candidate zone of nucleolus based on pathologist's decision. These results are shown in Figure 18 for nucleolus detection. In this figure the candidate regions detected by our algorithm are indicated by black line and expert diagnosed regions are shown by green line. The sensitivity and specificity for both nucleus and nucleolus candidate zone detection methods for all 100 images are shown in Table 2.

## 5. Conclusion

In this paper we introduced an algorithm for detection of candidate zone of nucleoli located in nuclei as a new tool for automatic exploring inside WBCs. This method is based on curvelet transform that is an appropriate tool for extraction and amplification of detailed information of cells such as nucleoli. 

The nucleolus candidate zone detection makes the blast cells diagnosis easier, and we tried to introduce a new feature to discriminate chromatins and nucleoli by means of gradient measure between candidate zones and texture of nucleus. Although the existence of nucleolus is a main symptom of blast cell, other features such as NCR, roundness factor, and absolute size of cells are also other important symptoms [1, 2, 28]. Based on this, the final results for automatic blast cell detection can be obtained by extraction of these features and using an appropriate classifier. 

## Figures and Tables

**Table 1:** Extracted nucleoli and their gradient value and delta measure.

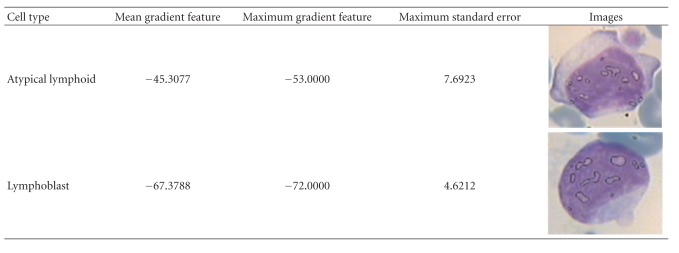

**Table 2:** Sensitivity and specificity of proposed algorithm in this paper for nucleus and candidate zone of nucleolus detection.

Parameter	Sensitivity	Specificity
Nucleus detection	90.4%	82.7%
Candidate zone detection	84.3%	80.2%
